# How Does Group Climate Foster or Hinder Employee Voice? A Cross-Level Examination

**DOI:** 10.3389/fpsyg.2021.609953

**Published:** 2021-10-12

**Authors:** Xiaoye Qian, Qian Li, Jue Wang, Shiyang Gong, Hao Zhou

**Affiliations:** ^1^Business School, Sichuan University, Chengdu, China; ^2^International Business School, Beijing Foreign Studies University, Beijing, China; ^3^School of International Business, Southwestern University of Finance and Economics, Chengdu, China; ^4^Business School, Beijing Normal University, Beijing, China

**Keywords:** group cooperation climate, group sanction climate, psychological capital, promotive voice, prohibitive voice, regulatory focus

## Abstract

Although empirical evidence has accumulated showing that group climate has a significant impact on employee voice, knowledge about how different types of climates may influence voice is limited. Drawing upon the theory of planned behavior, we develop and test a model that explains whether and how the two group climates, cooperation and sanction, differentially predict employee promotive and prohibitive voice. We test the hypotheses using data collected from a sample of 274 full-time employees nested in 58 workgroups across two time periods. The empirical results show that group climate predicts employee voice in different ways: Group cooperation climate has a positive effect on both types of employee voice, whereas group sanction climate shows a negative effect on promotive voice. Individuals’ psychological capital is a cross-level mediator in the relationship between group climate and employee voice. Employees’ prevention focus negatively moderates the relationship between psychological capital and employee voice. These results highlight the important effect of group climate on employee voice in organization and calls on managers to create a favorable environment to increase employees’ psychological capital and voice behaviors.

## Introduction

“*As we create a safe space for our people to speak up and speak out, where they can feel emboldened to point out both challenging areas and opportunities for new disruptions and innovations, our teams and organizations will thrive*.”

—Forbes (2020)

Employee voice refers to the discretionary communication of ideas, suggestions, concerns, or opinions about work-related issues with the intent to bring about improvement or changes (Morrison, 2011, 2014). In a hyperdynamic market, organizational success increasingly hinges on all employees providing valuable and timely suggestions. According to a survey of 464 business executives from 16 industry sectors in North America, Europe, and Asia Pacific, more than 90% agreed that organizational success depends on the voice from frontline workers (Business Wire, 2020). Toyota also provides a real-life example for this practice. When Toyota first took over the GM facility in California, it was highly unproductive. To solve the problem, Toyota launched the policy initiative of encouraging employees to speak up. As a result, Toyota ultimately implemented 80% of the employees’ suggestions, which led the plant to success (Emplify, 2020). Indeed, scholars have found that employee voice is associated with positive organizational performance (Ng and Feldman, 2012). Encouraging employee voice is an effective means to help organizations make high-quality decisions (LePine and Van Dyne, 1998; Nemeth et al., 2001), adapt to the ever-changing business environment (Dutton and Ashford, 1993; Floyd and Wooldridge, 1994), and foster and implement new ideas (Ng and Feldman, 2012). Empirical evidence also suggests that voice can improve team learning (Edmondson, 2003) and group performance (Lam and Mayer, 2014) and lead to other desired behaviors in organizations (MacKenzie et al., 2011). Given the prominent efficacy of voice, a substantive body of work has sought to identify its antecedents (for reviews, see Morrison, 2014; Chamberlin et al., 2017).

However, compared with abundant research on individual-level antecedents (e.g., Morrison, 2014; Chamberlin et al., 2017), literature on group-level antecedents has been largely limited so far (Peng and Wei, 2019). Existing research explores the effect of group size (LePine and Van Dyne, 1998), structure (Islam and Zyphur, 2005), support (Eisenberger et al., 1990), human resource management practices (Hu and Jiang, 2016; Wilkinson and Barry, 2016), and some specific climates (Morrison et al., 2011; Frazier and Bowler, 2015). Among these group-level antecedents, group climate, as an important voice-relevant contextual factor, has received growing yet still inadequate academic attention (Morrison and Milliken, 2000; Morrison et al., 2011; Hsiung and Tsai, 2017). Knowledge on the mechanism of how group climate affects employee voice is especially insufficient (Morrison et al., 2011; Frazier and Bowler, 2015).

To shed light on this issue, this study aims to answer the following questions: (1) what are the effects of the two important and prevailing group climates, cooperation and sanction, on employees’ voice behaviors? (2) What is the mechanism underlying such effects? (3) Will individuals’ characteristics, such as regulatory focus, moderate the relationship between group climate and voice behavior?

To address these questions, we adopt the theory of planned behavior (TPB; Ajzen, 1991) as the theoretical framework. Voice behavior is a planned behavior in nature. According to Liang et al. (2012), voice is a unique form of citizenship behavior because it is inherently challenging. Employees often have to conduct a cognitive calculation of costs and benefits before they decide to engage in any voice behavior (Liang et al., 2012; Morrison, 2014; Qian et al., 2020). Liang et al. (2012) clearly state that voice behavior should be considered as “an intentional, planned behavior” occurring in an interpersonal context. Therefore, the TPB fits voice behavior in nature and provides a solid theoretical foundation for understanding the relationship between group climate and employee voice.

Based on the TPB, we specifically propose the following. First, we propose that group cooperation and sanction climate may differently predict employees’ promotive (expression of new ideas or suggestions for improving the overall functioning of their work) and prohibitive voice (expression of concerns about work practices, incidents, or employee behaviors harmful to the organization), respectively (Liang et al., 2012). Different group climates may convey varied information that shapes people’s *attitude*, *subjective norm*, and *perceived behavioral control*, which are the three core constructs in the TPB that affect people’s intention to perform a certain behavior (Ajzen, 1991; Kozlowski, 2017).

Second, based on the TPB, we propose psychological capital as the mediator. Existing studies explore the mediating role of employees’ perception (e.g., perceived organizational support; Frazier and Bowler, 2015) and prosocial motivation (e.g., satisfaction and identification; Morrison et al., 2011) from the perspectives of social information processing theory and social exchange theory, but little attention has been paid to how group climate may shape one’s psychological dispositions, which consequently affect their cognitive evaluation of the risks and benefits relating to voice behaviors in a systematic manner. We, thus, argue that group climate may shape one’s positive psychological states (including self-efficacy, optimism, hope, and resilience; Luthans et al., 2007b), leading them to have more positive attitude toward and increased perceived control over voice behaviors.

Moreover, we propose regulatory focus as the moderator based on the TPB framework. Previous studies reveal that individuals’ personality traits are important variables that may change the cognitive process involved in TPB (Rhodes et al., 2006; Munir et al., 2019). Regulatory focus is such a trait that shapes how people perceive their environment and adjust their cognition and behaviors (Higgins and Spiegel, 2004; Lanaj et al., 2012). A promotion or prevention focus predisposes individuals to direct their psychological attention toward achieving positive outcomes or avoiding negative outcomes. Accordingly, we propose that individuals’ regulatory focus is an important person-based variable that moderates the relationship between psychological capital and voice behavior.

**Figure 1:**
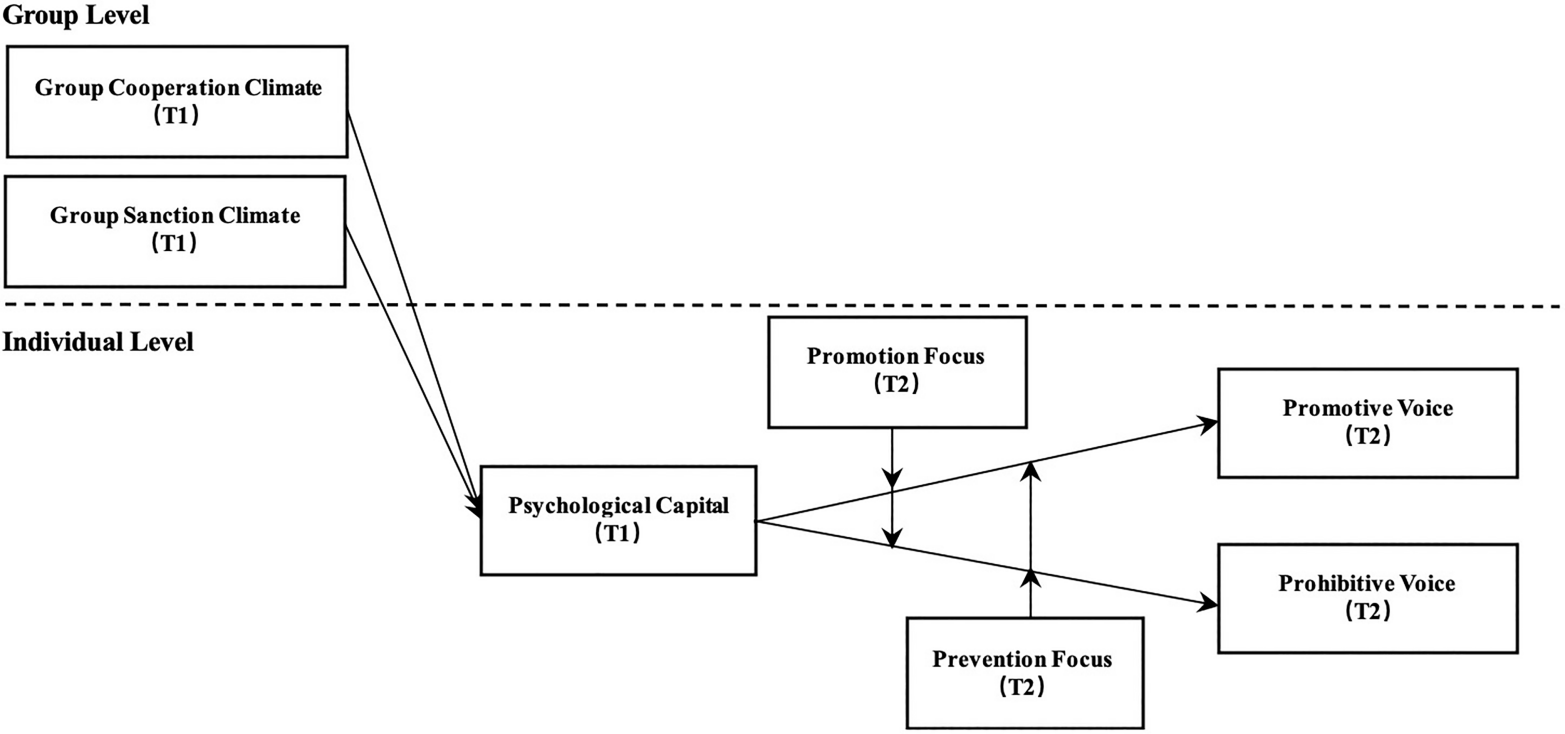
A cross-level mediation model of group climates on employee voice.

By examining these relationships, we make contributions to the literature. First, we extend research on the group-level antecedents of voice behavior. Based on the TPB, we identify two unexplored group climates, cooperation and sanction, which are important and prevailing in organizations and investigate their impacts on employees’ promotive and prohibitive voice. Second, we enhance theoretical understanding of the group climate–voice behavior relationship by suggesting psychological capital as the mediator and regulatory focus as the moderator. To our knowledge, previous research on *group climate–voice behavior* relationship has examined the mediation roles of individuals’ identification, satisfaction, and safety, but no one has discussed how group climate may shape one’s psychological dispositions, which consequently affect their cognitive evaluation of the risks and benefits relating to voice behaviors in a systematic manner. Our results prove that group climate may significantly affect one’s psychological capital and, hence, influence one’s voice. Also, we identify group members’ regulatory focus to be a moderator in the relationship between psychological capital and voice. This contributes to understanding the boundary conditions influencing the effect of psychological capital on voice behavior. Finally, our research enriches the psychological capital literature by introducing group climate as a cross-level predictor. The research model is shown in Figure 1.

This paper is organized as follows. First, we review previous literature and identify research gaps in existing studies. Second, based on the literature, we develop theoretical arguments and propose certain hypotheses regarding how group climate impacts employee voice, the mediating role of psychological capital, and the moderating role of regulatory focus. Third, we introduce our research design and empirical results. Then, we discuss the theoretical contributions and practical implications of our study. Finally, we address the limitations and future directions.

## Literature Review

### Group Climate and Voice Behavior

Group climate is a way in which a workgroup influences members’ psychology and behaviors at work. It is defined as “the shared perceptions and meaning group members attached to the events, policies, practices, and procedures they experience and the behaviors they observe getting rewarded, supported and expected in workgroups” (Schneider et al., 2013; Bollmann and Krings, 2016). Researchers study different types of organizational climates and find their impact on a variety of individual attitudes and behaviors. For example, the meta-analysis by Clarke (2010) demonstrates that a safety climate is highly related to individuals’ organizational commitment, job satisfaction, health and well-being, safety behavior, and occupational accidents. Bollmann and Krings (2016) find that a group compliance and relational climate significantly influences members’ counterproductive work behaviors. Besides this, group climates are proved to influence individuals’ work performance, innovation behavior, withdrawal behavior, and organizational citizenship behavior (Neal et al., 2000; Choi et al., 2003; Liao and Rupp, 2005; Zohar and Luria, 2005; Shanker et al., 2017).

In this study, we concentrate on two workplace group climates: cooperation and sanction climates. Group cooperation and sanction are the key group processes to predict work performance in the literature of social and industrial psychology (Varella et al., 2012). As Schneider et al. (2013) suggests, organizational processes might be practically studied and understood through a climate lens. For example, researchers conceptualize diverse group processes in climate terms, such as team change (Rafferty and Jimmieson, 2010), cooperative (Boerner and Freiherr von Streit, 2005), and trust climate (Brahm and Kunze, 2012). Studying these organizational issues from a climate perspective could yield new insights into the studies of workgroup and their correlations with varied outcomes.

James and James (1989) are among the first to posit that workgroup process–group cooperation is an important factor of psychological climate. It reflects “employees’ cognitive appraisal of the degrees to which the overall work environment is personally beneficial versus personally detrimental,” and the components of workgroup cooperation climates include *workgroup cooperation*, *responsibility for group effectiveness*, and *workgroup warmth and friendliness*. Varella et al. (2012) further emphasize two group processes: group cooperation and group sanction. They depict that, “*In groups embrace cooperation…group members typically believe it is safe to ask for support…increase the probability that group members will approach other members for support and engagement…and they are likely to respond positively. Sanction may have the opposite effect…group members concern about being ostracized and punished…fear of being exposed as inadequate or not living up to the group’s expectations…limit their interactions with other group members*.”

Based on previous studies, we define a group cooperation climate as “*the shared perceptions and beliefs held by group members that one should provide support, help, and feedback to each other to achieve group goals*.” A cooperation climate indicates that group members greatly appreciate each other’s help and contributions and are pretty tolerant of each other’s mistakes and inappropriate behaviors. Groups with such a climate provide high levels of safety and trust to members, and hence, they share the belief that mutual support is the pathway to achieve individual and group goals.

In contrast, a group sanction climate denotes a collective perception of ostracization and punishing among group members (Varella et al., 2012; Rudert et al., 2019). We define it as “*the shared perceptions and beliefs held by group members that one will be criticized, ostracized, and punished if he/she fails to meet the group expectations or nonconforms to the group norms*.” It indicates that group members should try to avoid undesirable outcomes and conform to the group norms. Under such a climate, group members may be reluctant to approach each other and easily become isolated for fear of exposing their inadequacy and weakness. Also, members may be conservative about risky behaviors because they are concerned about being ostracized and punished. This may result in low levels of safety and trust among group members and bring more accusation and blame among members (Costa et al., 2001).

Group climate is an important stimulus to shape individual behaviors (Kozlowski and Ilgen, 2006) because it conveys the information determining how group members recognize their behaviors (Kozlowski, 2017). Research shows that different group climates may have varied effects on voice behaviors (Kuenzi and Schminke, 2009). For example, Morrison and Milliken (2000) point out that, when employees encounter a climate of “silence” or “intolerance of dissent,” silence behaviors become a common pattern for employees. On the contrary, when the climate is conducive to giving advice, employees are inclined to give more suggestions (Morrison et al., 2011).

### TPB and Voice

Based on the TPB, an individual’s intention to perform a certain behavior is influenced by three factors: *attitude*, *subjective norm*, and *perceived behavioral control* (Ajzen, 1991). Attitude refers to whether a person has a favorable evaluation or appraisal of the behavior. The more positive attitude the person has toward the behavior, the more likely it is that the person may perform it. Subjective norm refers to an individual’s perceived social pressure to perform a behavior. If the individual feels that the important referent individuals or groups approve of performing a given behavior, the individual may have a stronger intention to engage in it. Perceived behavioral control refers to an individual’s perceived ease or difficulty of performing a behavior. It is determined by the presence of resources and opportunities, experiences of self and acquaintances, and anticipated impediments. The more resources individuals believe they possess and the fewer obstacles they anticipate, the greater perceived control they should have over the behavior.

To our knowledge, Liang et al. (2012) is among the first to explain employees’ involvement in voice behavior by adopting the TPB framework. After that, a growing literature has been following this theoretical perspective to study the influence of important contextual factors, such as authentic leadership (Xu et al., 2021) and high-commitment work systems (Zhang et al., 2019), on employees speaking up. However, among the limited studies exploring the group climate–voice relationship, this theoretical perspective has been neglected (Morrison et al., 2011; Frazier and Bowler, 2015). Previous studies mainly adopt social information processing theory and social exchange theory. They explore the mediating role of employees’ perception (e.g., perceived origination support; Frazier and Bowler, 2015) and prosocial motivation (e.g., satisfaction and identification; Morrison et al., 2011), yet little attention has been paid to psychological dispositions (e.g., self-efficacy, optimism, hope, and resilience).

### Psychological Capital

Psychological capital refers to an individual’s positive psychological state of development and is characterized by (1) self-efficacy: having confidence to take on and put in necessary effort to succeed at challenging tasks; (2) optimism: making a positive attribution about succeeding now and in the future; (3) hope: persevering toward goals and, when necessary, redirecting paths to goals to succeed; and (4) resiliency: when beset by problems and adversity, sustaining and bouncing back and even beyond to attain success (Luthans et al., 2007b).

Recent studies examine the mediating role of psychological capital in linking team-level predictors and individuals’ work outcomes (Newman et al., 2014). For example, Luthans et al. (2008) demonstrate that individuals’ psychological capital mediates the relationship between supportive climate and employee performance. Walumbwa et al. (2010) prove that individuals’ psychological capital is positively related to organizational citizenship behaviors. Also, there is growing evidence that a supportive organization environment facilitates individuals’ psychological capabilities, which leads to positive work attitudes, behaviors, and performance (Newman et al., 2014). A supportive work environment shapes one’s perception and beliefs and then increases individuals’ psychological capital. For example, Choi et al. (2003) demonstrate that a group supportive climate influences individuals’ accumulation of positive experience and efficacy, thus improving their psychological capital. Similarly, Luthans et al. (2008) find that a supportive climate had a positive impact on employees’ psychological capital.

### Regulatory Focus

Regulatory focus is a motivational principle that describes how people regulate themselves through two coexisting systems that cater to different needs during goal pursuit (Higgins and Spiegel, 2004; Lanaj et al., 2012; Song et al., 2020). Being promotion focused, people are motived to achieve growth and development needs, seek to attain goals associated with the ideal self, and are more sensitive to positive outcomes. Being prevention focused, people are responsive to security needs, seek to attain goals associated with the ought self, and are more sensitive to negative outcomes. The review by Lanaj et al. (2012) shows that individuals’ regulatory focus significantly influences job attitudes and performance, but the two systems may have different relationships with the outcomes. People with a promotion focus are more likely to engage in organizational citizenship, voice, and innovative behaviors to manage their work impressions and seek better development (Lin and Johnson, 2015). In contrast, prevention-focused employees are motived to follow rules and avoid making mistakes and, hence, engage in more safety behaviors.

By summarizing the relevant literature, we conclude that there are three research gaps in the current studies. First, research on group-level antecedents of voice behavior is largely limited. Most current research primarily examines the antecedents of voice behavior from the individual level, such as employees’ personalities, job attitudes, leadership styles, and leader–member exchange (Ng and Feldman, 2012; Morrison, 2014; Chamberlin et al., 2017; Zhou et al., 2020; Li and Tangirala, 2021). Literature on group-level antecedents of voice behavior is insufficient (Peng and Wei, 2019), especially on group climate, an important voice-relevant contextual factor (Morrison et al., 2011; Frazier and Bowler, 2015). Second, the climate–voice relationship needs further explorations by employing new theoretical perspectives. Current studies mainly adopt the social information and social exchange perspectives but have neglected the determinative role that cognitive processing plays in individuals’ voice decisions. Therefore, it is valuable to further explore the relationship based on the TPB. Third, there is a great need for studies of the underlying mechanism and boundary conditions for the group climate–voice behavior relationship.

## Hypotheses

### Group Climate and Employee Voice

According to the theory of planned behavior, we postulate that the group cooperation climate may be beneficial to increasing members’ voice behaviors. First, the group cooperation climate is conducive for employees to develop a positive attitude toward voice. Voice behavior is an organizational citizenship behavior when employees express their opinions and suggestions to improve work processes (Morrison, 2014). In a group with a cooperation climate, members share the belief that mutual support and help is the best way to achieve common goals. Individuals may highly value voice behavior in such groups because they think they are contributing to the group by sharing ideas. Accordingly, they may develop a favorable attitude toward voice because voice is closely associated with desirable group goals (Morrison, 2011; Qian et al., 2020). Second, a group cooperation climate provides positive feedback to group members’ voice behaviors. Unlike other organizational citizenship behaviors, voice behavior is unique in its challenging feature, which may hurt interpersonal relationships and change the status quo of a workgroup (Liang et al., 2012). Therefore, when members make decisions about whether to speak up in the group, they may consider the group norms. If they feel the social pressure to perform voice behaviors is low in the group, they are more likely to engage. A group with a cooperation climate conveys the social information that mutual help and feedback are encouraged (Boerner and Freiherr von Streit, 2005; Varella et al., 2012). When members share ideas and suggestions, they may feel that they are practicing the group norms, hence improving their normative beliefs about voice behaviors. Third, a group cooperation climate may increase individuals’ perceived behavioral control of voice behaviors. According to Ajzen (1991), one’s perceived behavioral control is determined by the *presence of resources*, *experiences*, and *anticipated obstacles*. In a group with a cooperation climate, members volunteer to help each other and actively provide feedback (Varella et al., 2012), so one may feel that one has access to adequate resources in the workgroup whenever one needs them. Also, it is quite possible that one may obtain a more favorable experience of performing voice behaviors in the group. Working in a team under a cooperation climate, employees may observe that coworkers always feel comfortable asking and providing suggestions to each other (James and James, 1989). They may even give suggestions to members who fall short of the group’s expectations. Accordingly, employees may learn from these positive experiences and feel more confident in making their own voice heard. Last, because the atmosphere of the team is collaborative and supportive, one may anticipate encountering fewer obstacles when they speak up in the workgroup. In sum, we propose the following:

*H1a*: A group cooperation climate is positively related to group members’ promotive voice.

*H1b*: A group cooperation climate is positively related to group members’ prohibitive voice.

In addition, we predict that a group sanction climate may decrease members’ voice behaviors. First, a group sanction climate may negatively impact one’s attitude toward voice behavior. A group with a sanction climate has a strong emphasis on punishing nonconforming members (Varella et al., 2012; Rudert et al., 2019). When members come up with a suggestion that may change the group status, even if it is valuable and beneficial to the group, they still risk being misunderstood as a “trouble-maker” and labeled as a “disobedient member” (Morrison, 2014). Therefore, members working in a sanction climate may have a negative attitude toward voice for fear of being misunderstood and may be conservative to speak up in the group. Second, a group cooperation climate may increase members’ perceived social pressure of performing voice behaviors. In a group with a sanction climate, members openly criticize others who do not follow the group norms and refuse to help nonconforming ones, which makes everyone in the group try their best to avoid undesirable outcomes (Costa et al., 2001; Rudert et al., 2019). If they speak their suggestions and concerns, such behaviors may easily be misinterpreted as “bossiness, unsolicited interference,” and an effort to undermine the creditability of others (Liang et al., 2012). In Eastern managerial contexts, harmony among team members is crucial (Kirkbride et al., 1991). Voice behavior may upset the interpersonal harmony and induce interpersonal tension within group (Morrison et al., 2011). Hence, in a sanction climate, members suffer from high levels of social pressure to perform voice behaviors. Third, a group sanction climate may decrease individuals’ perceived behavioral control of voice behaviors. As mentioned before, *the presence of resources*, *experiences*, and *anticipated obstacles* are the three determinants of perceived behavioral control (Ajzen, 1991). In a sanction climate, members know that coworkers will avoid helping nonconforming members. If they come up with suggestions to change the group status, they can hardly get resources within the group. Also, employees cannot learn positive experience and may observe that members are reluctant to approach coworkers and easily become isolated for fear of being punished by exposing weakness to others in the group (Varella et al., 2012; Rudert et al., 2019). Accordingly, employees may learn from this experience and be cautious about speaking up at work. Moreover, because the group atmosphere is punitive, employees have good reason to worry about the serious difficulties they may encounter if they are not careful enough in their acts of voice. Based on these arguments, we propose the following hypotheses:

*H2a*: A group sanction climate is negatively related to group members’ promotive voice.

*H2b*: A group sanction climate is negatively related to group members’ prohibitive voice.

### The Mediating Role of Psychological Capital

We propose that a group cooperation climate is positively related to individuals’ psychological capital. Group climate is an important stimulus to shape individual attitudes and behavior (Kozlowski and Ilgen, 2006) because it conveys information determining how group members recognize themselves and their behavior (Kozlowski, 2017). In groups that embrace cooperation, members receive support, encouragement, and recognition from group members. Therefore, they feel safe in the workgroup and do not have to worry about negative consequences if they encounter obstacles at work (James and James, 1989; Varella et al., 2012). Therefore, they are more confident at work and put more effort into challenging tasks (Bandura, 1997). Also, in a cooperative climate, employees receive different kinds of support from peers, including information, resources, and advice. When they encounter problems or feel frustrated at work, their coworkers are glad to help even without being asked. They may help cope with problems by leveraging their experience, attribute the problems or failures to external factors instead of personal abilities, and encourage peers to try different ways (Luthans et al., 2008). Accordingly, individuals may become more optimistic and hopeful about work (Choi et al., 2003). Moreover, a group with a cooperation climate is more tolerant of mistakes. Making mistakes at work is common. When people work in a cooperative group and fall short of the group’s expectations, their coworkers do not criticize and judge them. Instead, they provide feedback and support. The cooperative climate acts as a “soft cushion” for individuals to quickly bounce back after setbacks (Newman et al., 2014). In sum, we posit the following:

*H3a*: A group cooperation climate is positively related to group members’ psychological capital.

Next, we propose that a group sanction climate may have a negative impact on one’s psychological capital. The cooperation climate is associated with higher levels of support, help, and recognition, whereas the sanction climate emphasizes blame and punishment (Costa et al., 2001; Rudert et al., 2019). Members may perceive low levels of safety in the group and be worried about potential negative consequences at work. Being blamed or punished for undesired outcomes is like the “verbal persuasion” described by Bandura et al. (1996), providing information to individuals that “you are not competent” and harming their self-efficacy. Also, employees in a sanction climate may choose to maintain a distance from other members. Even if they need support, advice, or resources from others, they may be reluctant to approach others for the fear of exposing their weakness or bringing about negative interpersonal outcomes (Morrison, 2014). When members encounter problems or feel frustrated at work, they may not expect coworkers to help them voluntarily, and instead they may suffer from being criticized or ostracized (Costa et al., 2001). They may even attribute failures or mistakes to themselves for lacking personal abilities. Therefore, individuals are less optimistic about work (Luthans et al., 2008). Moreover, when a setback occurs or one gets stuck in a difficult situation, members in a sanction climate may find it hard to recover and stay persistent (lower levels of resilience) because they might face judgment and punishment and do not expect to get any encouragement or help. Based on these analyses, we propose the following:

*H3b*: A group sanction climate is negatively related to group members’ psychological capital.

Furthermore, we propose that group climate is associated with members’ psychological capital, which, in turn, leads to voice behavior. Recent studies examine the mediating role of psychological capital in linking team-level predictors and individuals’ work outcomes (Newman et al., 2014). For example, Luthans et al. (2008) demonstrate that individuals’ psychological capital mediates the relationship between a supportive climate and employee performance. Walumbwa et al. (2010) prove that individuals’ psychological capital is positively related to organizational citizenship behaviors. Based on this evidence, we propose that psychological capital is the underlying mechanism through which group climate influences voice behavior. First, we argue that individuals with high levels of self-efficacy and resiliency perceive high control over voice and are more likely to speak up. Self-efficacy, as a central aspect of psychological functioning (Taylor and Brown, 1988), greatly influences one’s perception of one’s capability in performing certain behaviors. Bandura (1997) argue that people with high self-efficacy have more positive expectations on the outcomes and, thus, behave more proactively. Accordingly, individuals with high self-efficacy are firm in their self-belief and are more confident in their abilities to control the outcomes of voice, thus being more active in speaking up at work (Morrison, 2014; Svendsen et al., 2016). Also, people with higher levels of resiliency can quickly recover from setbacks or negative feedback. Hence, they are less concerned about the potential difficulties of speaking up and believe they can surmount these problems. Moreover, group members with higher levels of optimism and hope are more likely to have a favorable evaluation of voice behaviors. They have a positive perspective on speaking up and persevering in trying different ways to achieve the desired goals. In line with previous research, we propose that individuals with high psychological capital are more likely to engage in voice behaviors instead of keeping silent (Newman et al., 2014). A group cooperation climate improves group members’ psychological capital and leads to higher involvement in speaking up. A group sanction climate lowers group members’ psychological capital, which results in less voice behaviors. In sum, we propose the following:

*H4a*: Psychological capital mediates the relationship between a group cooperation climate and group members’ promotive voice.

*H4b*: Psychological capital mediates the relationship between a group cooperation climate and group members’ prohibitive voice.

*H4c*: Psychological capital mediates the relationship between a group sanction climate and group members’ promotive voice.

*H4d*: Psychological capital mediates the relationship between a group sanction climate and group members’ prohibitive voice.

### The Moderating Role of Regulatory Focus

In line with previous literature, we expect that a promotion focus positively moderates the relationship between psychological capital and voice behavior. Specifically, when members are promotion focused, the positive effect of psychological capital on voice behavior is enhanced. They strive for growth and development, which matches the goal of voice behavior. Being promotion focused, people direct their psychological attention to getting positive outcomes (Higgins and Spiegel, 2004). They may become more confident and willing to contribute suggestions and ideas to the group with positive expectation of a better self and organization. Also, a promotion focus predisposes individuals to perceive more gains and consider less about losses (Higgins and Spiegel, 2004; Lanaj et al., 2012; Song et al., 2020). Accordingly, they are less concerned about the possible risks of voice and are more focused on the positive outcomes of voice, such as rewards, career opportunities, and improved self-concept. Hence, when people are promotion focused, the effect of their psychological capital on voice behaviors is enhanced. In contrast, we posit that a prevention focus negatively moderates the relationship between psychological capital and voice behavior. When people are prevention focused, they have higher levels of safety needs and are more sensitive to negative outcomes (Higgins and Spiegel, 2004). Given that the attributes of voice are challenging, prevention-focused employees may feel their psychological need for security is threatened and are, hence, less confident and optimistic to share ideas. Also, it is evidenced that people pay high social and career costs for voice behaviors (Koopmann et al., 2019). Because prevention-focused people are more sensitive to risks and negative outcomes at work, they are more likely to be conservative about voice behaviors. Therefore, the effect of psychological capital on voice behaviors is attenuated. We propose the following:

*H5a*: Promotion focus moderates the relationship between psychological capital and promotive voice such that, for individuals with promotion focus, the relationship between psychological capital and promotive voice is enhanced.

*H5b*: Promotion focus moderates the relationship between psychological capital and prohibitive voice such that, for individuals with promotion focus, the relationship between psychological capital and prohibitive voice is enhanced.

*H5c*: Prevention focus moderates the relationship between psychological capital and promotive behavior such that, for individuals with prevention focus, the relationship between psychological capital and promotive voice is attenuated.

*H5d*: Regulatory focus moderates the relationship between psychological capital and prohibitive behavior such that, for individuals with prevention focus, the relationship between psychological capital and prohibitive voice is attenuated.

## Materials and Methods

### Sample and Procedure

A total of 355 employees in 66 teams from Chinese organizations participated in our survey. Following the guidance and practice in organizational behavior research, to preclude common method bias, we collected the questionnaires in two different waves (Cooper et al., 2020; Li and Tangirala, 2021). At time 1, we measured the independent variable (group climate) and mediator variable (psychological capital). Two months later, at time 2, we measured the dependent variable (voice behavior) and moderator variable (regulatory focus) from the same participants. The HR department provided participants’ demographic information, including age, gender, education level, and job tenure. Participants were qualified only if they completed both phases of the study, which yielded 274 participants (77.18% response rate) for data analysis; 73% were female, and 55% had a bachelor’s degree or above. The average age was 37years old, and the average job tenure was 11years.

### Measures

All measures were translated into Chinese following the *translation-back translation* procedure (Brislin, 1970). A five-point Likert Scale (1=strongly disagree, 5=strongly agree) was adopted for all measures.

#### Group Climate (Time 1)

We adapted the Varella et al. (2012) scales to measure group cooperation (nine-item) and sanction climate (seven-item). Sample items include, “My coworkers and I feel comfortable asking for support from one another,” “My coworkers and I ostracize nonconforming members of the group.” Cronbach’s alpha was 0.93 for the cooperation climate and 0.92 for the sanction climate.

#### Psychological Capital (Time 1)

Participants rated their psychological capital using the 24-item scale by Luthans et al. (2007a). Cronbach’s alpha was 0.96.

#### Voice Behavior (Time 2)

Participants’ voice behaviors were measured by the 10-item scale developed by Liang et al. (2012). Cronbach’s alpha was 0.96 for the five-item promotive voice scale and 0.91 for the five-item prohibitive voice scale.

#### Regulatory Focus (Time 2)

Regulatory focus was assessed using a measure adapted from the one in the research of Lockwood et al. (2002). Cronbach’s alpha was 0.87 for promotion focus and 0.78 for prevention focus.

#### Control Variables

According to previous research, individuals’ demographic characteristics, including age (e.g., Wang et al., 2014), gender (e.g., Duan et al., 2017), education level (e.g., Zhou et al., 2020), and organizational tenure (e.g., Detert and Burris, 2007), have potential impact on their voice behaviors (Bidwell and Briscoe, 2009; Morrison, 2011; Liang et al., 2012). Therefore, we included these control variables to maintain consistency with previous studies with gender as a dummy variable (0=male; 1=female) and education level as a categorical variable (1=high school or below, 2=technical secondary school, 3=junior college, 4=college and above). At the group-level, we also controlled for team type (1=marketing, 2=logistics, 3=administration, 4=operation).

## Results

Descriptive statistics and correlations are reported in Table 1. Before verifying the hypotheses, we performed a confirmatory factor analysis to test the discriminant validity of the seven variables: group cooperation climate, group sanction climate, psychological capital, promotion focus, prevention focus, promotive voice, and prohibitive voice. As shown in Table 2, the hypothesized seven-factor model shows better fit [*χ*^2^=4621.193, *df*=1994, *χ*^2^/*df*=2.318, comparative fit index (CFI)=0.816, Tucker–Lewis index (TLI)=0.808, root mean square error of approximation (RMSEA)=0.069; standardized root mean square residual (SRMR)=0.079] than other models. The factor loadings of all items were above 0.55. Taken together, the results prove the discriminant and convergent validity of the studied variables.

**Table 1:** Mean, standard deviation, correlations, and reliabilities among studied variables.

Variable		*M*	*SD*	1	2	3	4	5	6	7	8	9
Individual-level												
1.	Gender	0.27	0.44	—								
2.	Age	37.05	8.83	0.20^***^	—							
3.	Education level	1.66	0.87	−0.10	−0.33^***^	—						
4.	Job tenure	11.35	8.71	0.03	0.68^***^	−0.22^***^	—					
5.	Psychological capital	3.65	0.61	0.03	0.02	0.04	−0.00	(0.96)				
6.	Promotive voice	3.18	0.84	0.11^*^	−0.07	0.12^*^	−0.03	0.27^***^	(0.96)			
7.	Prohibitive voice	3.35	0.71	0.10	0.03	0.10^*^	0.03	0.24^***^	0.78^***^	(0.91)		
8.	Promotion focus	3.53	0.57	0.01	−0.11^*^	−0.10	−0.04	0.32^***^	0.54^***^	0.60^***^	(0.87)	
9.	Prevention focus	3.10	0.64	0.05	−0.07	−0.00	−0.04	−0.10	0.27^***^	0.28^***^	0.46^***^	(0.78)
Team-level												
1.	Team type	2.06	0.83	—								
2.	Group cooperation climate	3.68	0.36	−0.19^***^	(0.93)							
3.	Group sanction climate	2.56	0.51	−0.05	−0.25^***^	(0.92)						

n_individual_=274; n_team_=58. Gender: 0=male and 1=female. Education level: 1=high school or below, 2=technical secondary school, 3=junior college, and 4=college and above. Team type: 1=marketing, 2=logistics, 3=administration, and 4=operation. Cronbach’s α values are shown in the brackets on the diagonal.

* *p*<0.1;

** *p*<0.05;

*** *p*<0.01.

**Table 2:** Results of confirmatory factor analyses.

	*χ*^2^	*df*	*χ*^2^*/df*	TLI	CFI	RMSEA	SRMR	∆*χ*^2^	∆*df*	*p*
7-factor model^a^	4621.193	1994	2.318	0.808	0.816	0.069	0.079			
6-factor model^b^	5946.458	2000	2.973	0.713	0.724	0.085	0.101	1325.265	6	<0.001
5-factor model^c^	6254.000	2005	3.119	0.692	0.703	0.088	0.103	307.542	5	<0.001
4-factor model^d^	6554.270	2009	3.263	0.671	0.682	0.091	0.104	300.270	4	<0.001
3-factor model^e^	8382.361	2012	4.166	0.539	0.554	0.107	0.163	1828.091	3	<0.001
2-factor model^f^	9385.678	2014	4.660	0.467	0.484	0.116	0.184	1003.317	2	<0.001
1-factor model^g^	10,860.426	2015	5.390	0.361	0.381	0.127	0.159	1474.748	1	<0.001

a Group cooperation climate, group sanction climate, promotive voice, prohibitive voice, promotion focus, prevention focus, and psychological capital.

b Group cooperation climate and group sanction climate combined, promotive voice, prohibitive voice, promotion focus, prevention focus, and psychological capital.

c Group cooperation climate and group sanction climate combined, promotive voice, prohibitive voice, promotion focus and prevention focus combined, and psychological capital.

d Group cooperation climate and group sanction climate combined, promotive voice and prohibitive voice combined, promotion focus and prevention focus combined, and psychological capital.

e Group cooperation climate, group sanction climate, promotive voice and prohibitive voice combined, promotion focus and prevention focus combined, and psychological capital.

f Group cooperation climate, group sanction climate, promotive voice and prohibitive voice combined, psychological capital, promotion focus, and prevention focus combined.

g All seven variables combined.

To justify the aggregation of group climate, we checked the agreement among team members and the variance between teams. We calculated r*_wg_*, intraclass correlation coefficient (ICC; 1), and ICC (2) as indicators of within-group agreement, interrater reliability, and group-mean reliability (Bliese, 2000). The r*_wg_* of the group cooperation and sanction climates was 0.97 and 0.93, so both passed the standard threshold of 0.7. The ICCs for group cooperation climate were ICC (1)=0.17, ICC (2)=0.89, and the ICCs for group sanction climate were ICC (1)=0.18, ICC (2)=0.82. Both ICCs were above the recommended cutoffs, justifying the aggregation of group climate.

We used hierarchical linear modeling (HLM) in Stata14 to test our hypotheses. According to the suggestions of Liao and Chuang (2007), we mean-centered both individual- and team-level variables before further analysis (Hofmann and Gavin, 1998; Enders and Tofighi, 2007). The regression results are in Table 3. First, a null model was tested without predictor variables. We estimated the between-team variance in voice behavior by examining the group-level residual variance of the intercept (*τ*) and individual-level residual variance (*σ*^2^) and by calculating ICC (1). Results show that, for promotive voice behavior, *τ*=0.08, *p*<0.05, *σ*^2^=0.63, and ICC (1)=0.11, indicating that 11% of variance in promotive voice resided between teams. For prohibitive voice behavior, *τ*=0.05, *p*<0.05, *σ*^2^=0.46, and ICC (1)=0.10, indicating that 10% of variance in prohibitive voice behavior resided between teams. Therefore, it provided the evidence to do further cross-level investigation (Enders and Tofighi, 2007).

**Table 3:** HLM results of the hypothesized relationships.

Variable	Promotive voice							Prohibitive voice						Psychological capital	
	Null model	M1	M2	M3	M4	M5	M6	Null model	M7	M8	M9	M10	M11	M12	M13
Intercept	0.02	0.17	0.21	0.22	0.22	−0.33	−0.41	0.02	−0.28	−0.27	−0.22	−0.95	−1.00	−0.09	0.02
Individual-level															
Gender		0.22^*^	0.22^*^	0.24^**^	0.23^**^	0.20^**^	0.20^*^		0.12	0.12	0.13	0.10	0.10	−0.01	0.04
Age		−0.01	−0.01	−0.01	−0.01	−0.002	−0.01		0.00	0.00	0.01	0.01^*^	0.01	0.003	0.01
Education level		0.13^**^	0.11^*^	0.12^*^	0.11^*^	0.06	−0.10		0.15^***^	0.14^***^	0.14^*^ ^*^	0.10^**^	0.13^**^	0.04	0.03
Job tenure		0.01	0.01	0.01	0.01	0.002	0.01		0.001	0.002	0.001	−0.001	0.003	0.002	−0.003
Psychological capital (M)			0.34^***^		0.35^***^	0.13^*^	0.42^***^			0.24^***^		0.04	0.32^***^		
Promotion focus (W1)						0.77^***^						0.74^***^			
Prevention focus (W2)							0.46^***^						0.40^***^		
M×W1						0.03						0.04			
M× W2							−0.22^**^						−0.16^*^		
Team-level															
Team type		0.10	0.09	0.06	0.08	0.05	0.10		0.14^**^	0.14^**^	0.11^*^	0.10^**^	0.14^**^	0.01	−0.06
Cooperation climate		0.37^**^	0.15						0.36^***^	0.20				0.62^***^	
Sanction climate				−0.21^*^	−0.12						−0.08				−0.25^***^
Variance decomposition															
Variance within group (σ^2^)	0.63	0.63	0.59	0.62	0.58	0.41	0.49	0.46	0.46	0.43	0.45	0.28	0.36	0.32	0.34
Variance between group (τ)	0.08	0.03	0.03	0.05	0.03	0.06	0.07	0.05	0.01	0.02	0.04	0.03	0.04	0.00	0.02
Log likelihood	−337.16	−330.40	−322.27	−331.37	−322.08	−281.44	−304.61	−293.42	−330.42	−279.64	−288.28	−233.94	−261.76	−234.33	−246.97

n_individual_=274; n_team_=58.

* *p*<0.1;

** *p*<0.05;

*** *p*<0.01.

Hypotheses 1a and 1b predict that group cooperation climate is positively related to employees’ promotive and prohibitive voices. As shown in Models 1 and 7 of Table 3, group cooperation climate had significantly positive relationships with group members’ promotive (γ=0.37, *p*<0.05) and prohibitive voice (γ=0.36, *p*<0.01). Therefore, Hypotheses 1a and 1b were supported.

Hypotheses 2a and 2b predict that group sanction climate is negatively related to members’ promotive and prohibitive voices. As shown in Models 3 and 9 of Table 3, the relationship between group sanction climate and promotive voice is significantly negative (*γ*=−0.21, *p*<0.1), but the relationship between group sanction climate and prohibitive voice was negative though not significant. Therefore, Hypothesis 2a is supported, and Hypothesis 2b is not supported.

Hypotheses 3a and 3b predict the cross-level direct effect of group climate on members’ psychological capital. As displayed in Models 12 and 13 of Table 3, group cooperation climate was positively related to psychological capital (*γ*=0.62, *p*<0.01), and group sanction climate was negatively related to psychological capital (*γ*=−0.25, *p*<0.01). Thus, Hypotheses 3a and 3b are supported.

Hypothesis 4 predicts the mediating role of psychological capital in the relationship between group climate and voice behavior. We tested this hypothesis by following the procedures for cross-level mediation analysis suggested by Preacher et al. (2010) and the analysis strategy for testing mediation hypotheses by Baron and Kenny (1986). First, in Hypotheses 1 and 2, we found that the independent variable (group climate as X) was significantly related to the dependent variable (group members’ voice behavior as Y). In Hypothesis 3, the independent variable (group climate as X) was shown to have significant relationships with the mediator (group members’ psychological capital as M). Thus, the first two conditions of the mediation test (X→Y, X→M) were satisfied (Baron and Kenny, 1986). Then, the final step was to regress group members’ voice behavior on both group climate and psychological capital. As shown in Models 2, 4, and 8 of Table 3, when psychological capital is included, the effects of group climate on voice behavior became insignificant, but the effects of psychological capital are still significant for both promotive voice (*γ*=0.34, *p*<0.01 in Model 2; *γ*=0.35, *p*<0.01 in Model 4) and prohibitive voice (*γ*=0.24, *p*<0.01 in Model 6).

To further assess the significance of mediation, we conducted bootstrapping analysis (Preacher and Selig, 2012). The results show that the indirect effects of group cooperation climate on promotive [95% CI=(0.177, 0.503), not containing zero] and prohibitive voice [95% CI=(0.098, 0.377), not containing zero] through psychological capital are both significant. Similarly, the indirect effect of group sanction climate on promotive voice through psychological capital [95% CI=(0.193, 0.505), not containing zero] is also significant. Thus, Hypotheses 4a–c are supported, and Hypothesis 4d is not supported.

Hypothesis 5 predicts the moderating role of group members’ regulatory focus in the relationship between psychological capital and voice behavior. Specifically, we posit that a promotion focus would strengthen the *psychological capital–voice behavior* relationship, and the prevention focus would weaken the relationship. As shown in Models 6 and 11 of Table 3, the correlations between psychological capital and prevention focus were negative and significant (for promotion voice, *γ*=−0.22, *p*<0.05; for prohibitive voice, *γ*=−0.16, *p*<0.1), which suggests that, for group members with a higher level of prevention focus, the effect of psychological capital on voice behavior is attenuated. However, the interactions between psychological capital and promotion focus were positive yet insignificant (as shown in Models 5 and 10 of Table 3). Therefore, Hypotheses 5c and 5d are supported, and Hypotheses 5a and 5b are not supported.

## Concluding Remarks

Drawing on TPB, we set out to examine how two group climates, cooperation and sanction, would uniquely and differentially predict group members’ promotive and prohibitive voice. We employed a two-wave panel design to test our hypotheses. The empirical results show that the two group climates predict employee voice behaviors in different ways. Group cooperation climate is positively related to both promotive and prohibitive voice. When group members get considerable support and help from the group, they may have positive attitudes and perceive higher behavioral control over voice behaviors, and thus would actively engage in idea contribution. In contrast, group sanction climate shows a negative pattern. Group sanction climate has a significantly negative impact on promotive voice, and its impact on prohibitive voice is negative but not significant. This may be because, in an Asian cultural context, people highly value group harmony and may overestimate the risk of prohibitive voice. Unless they perceive a comparatively safe and supportive group climate, they are reluctant to engage in any prohibitive voice behavior.

Moreover, we find that a member’s psychological capital is a cross-level mediator in the relationship between group climate and voice behavior. A group cooperation climate significantly increases members’ psychological capital and, hence, promotes their promotive and prohibitive voice. Group sanction climate negatively impacts members’ psychological capital, which leads to less promotive voice. Also, the results show that people’s regulatory focus is a moderator in the relationship between psychological capital and voice behavior. When people were prevention focused, the effect of psychological capital on voice behavior is attenuated. However, the moderating role of promotion focus is not supported, maybe because our participants are from China, where interpersonal relationships and impression management are highly valued. Even though they are promotion focused, they cannot completely overlook the risk of voice behavior, which eliminate the positive moderation effect of promotion focus.

### Theoretical Implications

Our findings contribute to the understanding of employee voice in groups in three ways. First, our study extends voice literature by illustrating the cross-level effects of group climates on employee voice. In particular, we introduce two new group climates – cooperation and sanction – as the antecedents of voice behavior. As Morrison and Milliken (2000) point out, group climate is an important factor that influences voice behavior. Although several group climates are discussed in previous research (Morrison et al., 2011; Frazier and Bowler, 2015), little attention has been paid to organizational processes through a climate lens. Group cooperation and sanction are the key group processes to predict work performance in the literature of social and industrial psychology (Varella et al., 2012). As Schneider et al. (2013) suggest, researchers should conceptualize diverse group processes in climate terms, which would yield new insights into the studies of climate and work outcomes. Therefore, in line with Varella et al. (2012), we introduce the group cooperation and sanction climates as the new antecedents of employee voice on the group level. Drawing on the TPB, we show that the group cooperation climate is positively related to employee voice, and the group sanction climate is negatively related to employee voice.

Second, we enhance the theoretical understanding of group climate–voice behavior relationship *via* psychological capital. We find that group members’ psychological capital is a cross-level mediator in the relationship between group climate and voice behavior. To our knowledge, previous research examines the mediation roles of individuals’ identification, satisfaction, and safety in the group climate–voice behavior literature, but no one has discussed members’ psychological capital (Morrison et al., 2011). Our results prove that group climate may significantly shape one’s psychological capital, which includes self-efficacy, hope, optimism, and resiliency, and, hence, influence their voice. Also, we find that group members’ regulatory focus is a moderator in the relationship between psychological capital and voice behavior. The findings contribute to understanding the boundary conditions influencing the effects of psychological capital on voice behavior.

Third, our research contributes to the psychological capital literature by introducing group climate as a cross-level predictor. For the group-level antecedent of psychological capital, prior work mainly focuses on the behaviors of group leaders, such as ethical leadership, shared leadership, and abusive supervision (Newman et al., 2014). To our knowledge, little empirical attention has been paid to the role of group climate in shaping one’s psychological capital. In this research, we find that group cooperation climate may significantly improve members’ psychological capital, which makes them more confident and optimistic to engage in challenging behaviors, and the group sanction climate may diminish their psychological capital and make them more conservative about voice behaviors.

### Practical Implications

Our findings also provide several practical implications. First, our research calls on managers to realize the great value of a favorable group climate in encouraging employee voice. Findings of the current study suggest that the cooperation climate encourages employees’ voice behaviors, and the sanction climate dissuades them from speaking up. Therefore, group leaders need to foster a safe environment in which employee input is valued and also try to avoid discouraging or even punishing members from speaking up. Second, managers can learn from our study that group climate may influence employees’ psychological capital and affect their cognitive evaluation of voice behaviors. Thus, managers should advocate guidelines that encourage timely feedback and support from group leaders and coworkers to members in need of resources. Third, we suggest that organizations highly value employees’ regulatory focus and provide them with appropriate support. For members who are prevention focused, it is better for organizations and group leaders to carefully address their concerns and avoid giving negative feedback. Once the employees are less sensitive to the risks, they may put forward more ideas and suggestions, and contribute more to the group.

### Limitations and Directions for Future Research

Our study has several limitations that can be addressed by future research. First, to eliminate common method bias, we collect data from employees at two different times in this study. For future research, we encourage researchers to collect data from multiple recourses to reduce self-report bias and improve the causal inference. Second, workgroups in this study are from the same industry, which may limit the generalization of the results. Although the surveyed groups cover a wide array of departments and tasks, it is valuable to apply our sample to other different industries. Third, our model is tested in the Chinese context. Chinese culture and values may make people more sensitive to prohibitive voice. We recommend that future research systematically examine our conclusions in different cultural contexts.

## Data Availability Statement

The data used in this study are available upon request to the corresponding author.

## Ethics Statement

The studies involving human participants were reviewed and approved by Beijing Foreign Studies University. The patients/participants provided their written informed consent to participate in this study.

## Author Contributions

XQ, QL, and HZ contributed to the research concept and design. XQ and QL collected and analyzed the data. XQ, QL, and SG drafted the manuscript. HZ and JW provided the critical revisions. All authors contributed to the article and approved the submitted version.

## Funding

We gratefully acknowledge financial support from the National Natural Science Foundation of China (Nos. 71872117, 72072014, 71702010, 71972040, 71602033, and 71872119), Beijing Foreign Studies University Young Faculty Research Fund (No. 2021JT001), and Double First-Class Project (Nos. SYL2020ZX012 and sksyl201703).

## Conflict of Interest

The authors declare that the research was conducted in the absence of any commercial or financial relationships that could be construed as a potential conflict of interest.

## Publisher’s Note

All claims expressed in this article are solely those of the authors and do not necessarily represent those of their affiliated organizations, or those of the publisher, the editors and the reviewers. Any product that may be evaluated in this article, or claim that may be made by its manufacturer, is not guaranteed or endorsed by the publisher.
